# A Rapid and Cheap Methodology for CRISPR/Cas9 Zebrafish Mutant Screening

**DOI:** 10.1007/s12033-015-9905-y

**Published:** 2015-12-16

**Authors:** Ylenia D’Agostino, Annamaria Locascio, Filomena Ristoratore, Paolo Sordino, Antonietta Spagnuolo, Marco Borra, Salvatore D’Aniello

**Affiliations:** Biology and Evolution of Marine Organisms (BEOM), Stazione Zoologica Anton Dohrn, Villa Comunale, 80121 Naples, Italy; Research Infrastructures for Marine Biological Resources (RIMAR), Stazione Zoologica Anton Dohrn, Villa Comunale, 80121 Naples, Italy

**Keywords:** Zebrafish *knock*-*out* mutants, CRISPR/Cas9, qPCR, Derivative melting curve, Mutation screening

## Abstract

The introduction of new genome editing tools such as ZFNs, TALENs and, more recently, the CRISPR/Cas9 system, has greatly expanded the ability to *knock*-*out* genes in different animal models, including zebrafish. However, time and costs required for the screening of a huge number of animals, aimed to identify first founder fishes (F0), and then carriers (F1) are still a bottleneck. Currently, high-resolution melting (HRM) analysis is the most efficient technology for large-scale InDels detection, but the very expensive equipment demanded for its application may represent a limitation for research laboratories. Here, we propose a rapid and cheap method for high-throughput genotyping that displays efficiency rate similar to the HRM. In fact, using a common ViiA™7 real-time PCR system and optimizing the parameters of the melting analysis, we demonstrated that it is possible to discriminate between the mutant and the wild type melting curves. Due to its simplicity, rapidity and cheapness, our method can be used as a preliminary one-step approach for massive screening, in order to restrict the scope at a limited number of embryos and to focus merely on them for the next sequencing step, necessary for the exact sequence identification of the induced mutation. Moreover, thanks to its versatility, this simple approach can be readily adapted to the detection of any kind of genome editing approach directed to genes or regulatory regions and can be applied to many other animal models.

**Electronic supplementary material:**

The online version of this article (doi:10.1007/s12033-015-9905-y) contains supplementary material, which is available to authorized users.

## Introduction

In the last 10 years, morpholino anti-sense oligonucleotides have been the most common *knock*-*down* technique used in zebrafish, as well as in many other organisms [1]. However, to better understand the function of a given gene, especially during adulthood, hereditable genetic mutations are desirable [2]. In order to induce site-specific mutations, genome editing tools have become fundamental for reverse genetics studies and loss-of-function approaches in different animal models, including zebrafish.

Zinc-finger nucleases (ZFNs) and transcription activator-like effector nucleases (TALENs) are the first examples, in which endonuclease catalytic domains are connected to DNA-binding proteins for the purpose of causing DNA double-stranded breaks (DSB) in a specific genomic locus [3]. Once the DSB is determined, the endogenous error-prone Non-Homologous End-Joining system (NHEJ) repairs the damage in absence of a template, leading to random insertion or deletion (InDels) at the cut site [4].

More recently, the CRISPR (Clustered Regularly Interspaced Short Palindromic Repeats)/Cas9 system has been introduced as a new class of genome engineering tool, also for organisms with a genome difficult to edit like the one of zebrafish [3, 5]. This system is naturally present in eubacteria and archaea, which use it as an adaptive immune defence against exogenous molecules of DNA, such as a viral infection [6]. The type II from *Streptococcus pyogenes* is one of the best characterized CRISPR system [7], and actually it can be reproduced in vitro through the synthesis of a guide RNA (gRNA) and the mRNA encoding Cas9, which are co-injected in one-cell stage embryos.

The genomic target site must be 20 bp long, immediately upstream a Protospacer Adjacent Motif (PAM) 5′-NGG/NCC [8]. Simplistically, the gRNA binds to the target sequence following the Watson–Crick base pairing and leads to specific nuclease cleavage in the gene of interest. Then, the repairing of the break by the NHEJ system results in unpredictable genetic mutations.

Compared to ZFNs and TALENs, the gRNA of the CRISPR/Cas9 system is the unique customized element that needs to be designed for each target gene, thus consistently reducing costs and working times. The bioinformatic analysis to identify the best target region, the molecular cloning steps and the synthesis of gRNA and Cas9 mRNA are greatly simplified in the CRISPR/Cas9 system, thanks also to the easy access to plasmid repositories, such as Addgene [9]. Nevertheless, the difficulties connected to the genotyping aiming at the generation of a stable *knock*-*out* line still remain the bottleneck of the entire process.

The injected zebrafish embryos (F0) in fact are mosaics, therefore induced mutations in their germline must be detected through a second screening-step in the F1 progeny. Moreover, if homozygous mutation is required, the F2 progeny must be generated, grown and genotyped, in order to identify two heterozygous fishes carrying the same mutation which must be crossed to reach the F3 progeny [10]. Alternatively to shorten the breeding time, heterozygous F1 fishes carrying the same mutation can be crossed to obtain the homozygous mutant in the next generation (F2) [11].

Several approaches can be used to screen mutant fish generations produced by the CRISPR/Cas9 system, with different peculiarities in related costs, needed time, and accuracy. To avoid that this step becomes a limiting factor for this powerful system, it is important to critically consider the characteristics of different methodologies and to choose the appropriate one before undertaking a genome editing project. Nevertheless, the complete process to obtain the homozygous *knock*-*out* fish requires a combination of more than one screening techniques among: 1. Locus DNA sequencing; 2. Fluorescent PCR; 3. PAGE-based genotyping approach; 4. T7 endonuclease I assay and 5. High-Resolution Melting (HRM).

### Locus DNA Sequencing

DNA sequencing of the genomic locus of interest is the most informative approach since it implies the exact determination of the putative produced mutation. On the other hand, this technique is quite time-consuming, requires access to high-complex instrumentation and methods like automated capillary electrophoresis sequencer, and has not trivial costs. Moreover, the exact determination of the induced genomic perturbation is often not needed in initial steps of homozygous mutant screening, while the goal is the selection of those embryos that later will be crossed to obtain the mutant fish line.

### Fluorescent PCR

This technique follows the same basic principles of a common PCR; however, in this case, the primers are labelled with fluorescent markers in order to make the system more sensitive. PCR amplicons are then separated and analysed using an ultrasensitive system fluorescent DNA sequencer, commonly known as a Genescanner, instead of the agarose gel. The different dyes used to label the primers allow discriminating between two PCR products with an accuracy of 1 or 2 bp [12].

### PAGE-Based Genotyping Methodology

In this approach, the PCR-amplified genomic regions spanning the mutagenesis site undergo a brief denaturation and annealing cycle. Then, PCR fragments from genetically modified individuals, which contain a mixture of InDel mutations and wild type (wt) alleles, will form heteroduplex and homoduplex DNAs. Due to the existence of an open angle between matched and mismatched DNA strands caused by InDel mutations, heteroduplex DNA generally migrate at a significantly slower rate than homoduplex DNA in a native Polyacrylamide Gel Electrophoresis (PAGE), thus making it a useful tool to screen founders harbouring mutations [13]. However, this is not a high-throughput approach, it is time-consuming and it does not provide any exact information about the mutations, although it is affordable in terms of feasibility and costs.

### T7 Endonuclease I Assay

PCR approaches can be usefully applied to screen mutations produced by the CRISPR/Cas9 system. In the T7 endonuclease I assay (T7E1 is a mismatch-specific DNA endonuclease), the mutated target region is PCR amplified and then digested by specific restriction enzymes. Thus, this approach permits to determine the genotype by revealing the different size of digested and undigested PCR fragments on agarose gel electrophoresis [14]. Also in this case, this is not a large-scale approach and it does not give precise information about the mutations.

### HRM-Based Assay

High-resolution melting analysis approach can be successfully used to screen mutagenesis in the injected generation (F_0_), which results in a mixture of wt and mutant PCR products, and melts at different (lower) temperatures compared to not injected control embryos [15]. In this approach, the melting temperature of the PCR-amplified genomic regions spanning the mutated site is analysed in high resolution during a dissociation curve temperature profile. However, this approach is quite expensive given the high cost of the HRM qPCR instruments and the specific software required for data analysis.

## Materials and Methods

### PCR, Cloning and Sequencing

Genomic DNA (gDNA) was extracted by caudal fin clipping. PCR-amplified fragments of the locus of interest were cloned in pGEM-T Easy vector (Promega). PCR amplification reactions were conducted in final volumes of 25 μl containing PCR reaction buffer with MgCl_2_ (Roche), about 70 ng of gDNA, 2.5 μM of Forward and Reverse primers, dNTP (2 mM) and GoTaq DNA Polymerase (0.25 U/μl) (Promega). The gDNA amplification was performed with 28 cycles at the annealing temperature of 55 °C. The length of DNA fragments was checked on 1 % agarose gel. Sequence reactions were obtained with the BigDye Terminator Cycle Sequencing technology (Applied Biosystems) and purified in automation using the Agencourt CleanSEQ Dye terminator removal Kit (Agencourt Bioscience Corporation) and a robotic station Biomek FX (Beckman Coulter). DNA products were analysed on an Automated Capillary Electrophoresis Sequencer 3730 DNA Analyzer (Applied Biosystems).

### qPCR

Real-time PCR (qPCR) amplification was performed with undiluted gDNA in a reaction containing a final concentration of 0.7 μM for each primer and Fast SYBR Green Master mix with ROX (Applied Biosystems) in 10 μl total volume. Reactions were run in a ViiA™ 7 Real-Time PCR System (Applied Biosystems). The cycling condition was: 95 °C for 20 s, 40 cycles at 95 °C for 1 s, 60 °C for 20 s, 95 °C for 15 s, 60 °C 1 min, and a gradient from 60 °C to 95 °C with a continuous detection at 0.015 °C/sec increment for 15 min. The results were analysed using the ViiA™ 7 Software and exported into Microsoft Excel for further analysis. Every sample was processed with technical triplicates.

### CRISPR/Cas9

The CRISPR/Cas9 approach was performed following the protocol from the Chen and Wente laboratories, as described in [16]. The engineered vectors were provided from Addgene.

### Insert-Primers Design

The genomic target site was identified using a publicly available web tool (http://crispr.mit.edu/). The complement and reverse insert-primers were designed as standard primers (Sigma), suspended in TE (10 mM Tris–HCl, 0.1 mM EDTA) to generate a 100 µM stock solution. The two primers (2 µl of each stock) were then annealed in NEB buffer solution using an incubator.

### Synthesis of gRNA and Cas9 mRNA

To prepare the gRNAs (guide RNA), the pT7-gRNA vector (Addgene) was linearized by BamHI digestion and purified using a QIAprep column (Qiagen). The DNA template was directionally transcribed in vitro using the MEGAshortscript T7 kit (Ambion-Invitrogen) and purified with the mirVana miRNA isolation kit (Ambion-Invitrogen).

To produce the capped nls-zCas9-nls mRNA, the pT3TS-nls-zCas-nls vector (Addgene) was linearized by Xba I digestion and purified using a QIAprep column. The DNA template was directionally transcribed in vitro using the mMESSAGE mMachine T3 kit (Ambion-Invitrogen) and purified with the RNeasy Mini kit (Qiagen).

### Microinjection into Zebrafish Zygote and T7 Endonuclease I

One ml of a mixed solution containing gRNA (80 ng/µl) and purified Cas9 mRNA (150 ng/µl) was microinjected into one-cell stage zebrafish embryos. The efficiency of mutagenesis was assessed using the T7 endonuclease I assay (New England BioLabs), following the manufacturer’s instructions (data not showed).

## Results and Discussion

More and more often, genome manipulation is becoming a widespread approach for reverse genetics studies in every field of research. In particular, genome engineering has been revolutionized by the introduction of new editing tools such as ZFNs, TALENs and, more recently, the CRISPR/Cas9 system.

However, as the ability to generate mutated animals increases, the screening of mutations is becoming a bottleneck. So far, many different techniques such as direct sequencing, fluorescent PCR, T7 endonuclease I assay, PAGE and HRM have been described as efficient methods for the detection of InDels in the *locus* of interest but at the same time they are not “within everyone’s reach”.

Surely, the most informative method is the direct sequencing by Sanger chromatography; however, this approach implies several steps such as fin clipping, genomic DNA extraction, PCR amplification of the target region, cloning and plasmid purification, which are cost-prohibitive for massive screenings.

For this reason, the PAGE and T7 endonuclease I assays, being affordable in terms of cost, are becoming the most used approaches, but they present some limitations such as time-consuming steps and false positive. A valid alternative, in terms of reliability and run-time, is represented by the HRM technique that is currently the most efficient technology for large-scale InDels detection. Indeed, the HRM protocol is simple and rapid once the proper equipment is present in the laboratory; however, not all the laboratories can afford the costs of such an expensive instrument. As a consequence, the major effort is now to develop a cheap and efficient strategy for high-throughput mutants screening that can be accessible to everyone.

We here propose a new approach for genotyping with an efficiency rate similar to HRM technique, but much cheaper in terms of total costs, by changing some parameters of a common ViiA™ 7 Real-Time PCR System for qPCR. After several trials, we optimized the reaction conditions focusing on the melting analysis step. The amplification conditions were not modified since this step is only functional to produce the amplified molecules that will be analysed by the melting profile. The melting analysis parameters were optimized to obtain the higher possible resolution and reproducibility of the detection. In particular, the ramp increment was increased up to 0.05 °C/s with a continuous fluorescence detection. The increase of the ramp rate resulted in a more accurate description of the dissociation profile from the analysed sample types because of faster dissociation dynamics of the molecules. In addition, we optimized the working protocol, thanks to the 384 well format of the plate, which allows reducing the reaction volume (total volume 10 µl). This implies the use of only 5 µl of Fast SYBR Green Master mix per sample, thus appreciably reducing the costs.

With these optimized parameters, it is possible to screen different fish generations required to reach a stable *knock*-*out* line: F0 (mosaic fishes generated by eggs microinjection), F1 (generated by crossing wt with F0 fishes, carrying mutations in the germinal line, resulting in a population of different heterozygous fishes), F2 (generated by crossing wt with F1 fishes, carrying the desirable mutation), and finally F3 (generated by crossing two F2 heterozygote fishes with the same selected mutation).

**Fig. 1:**
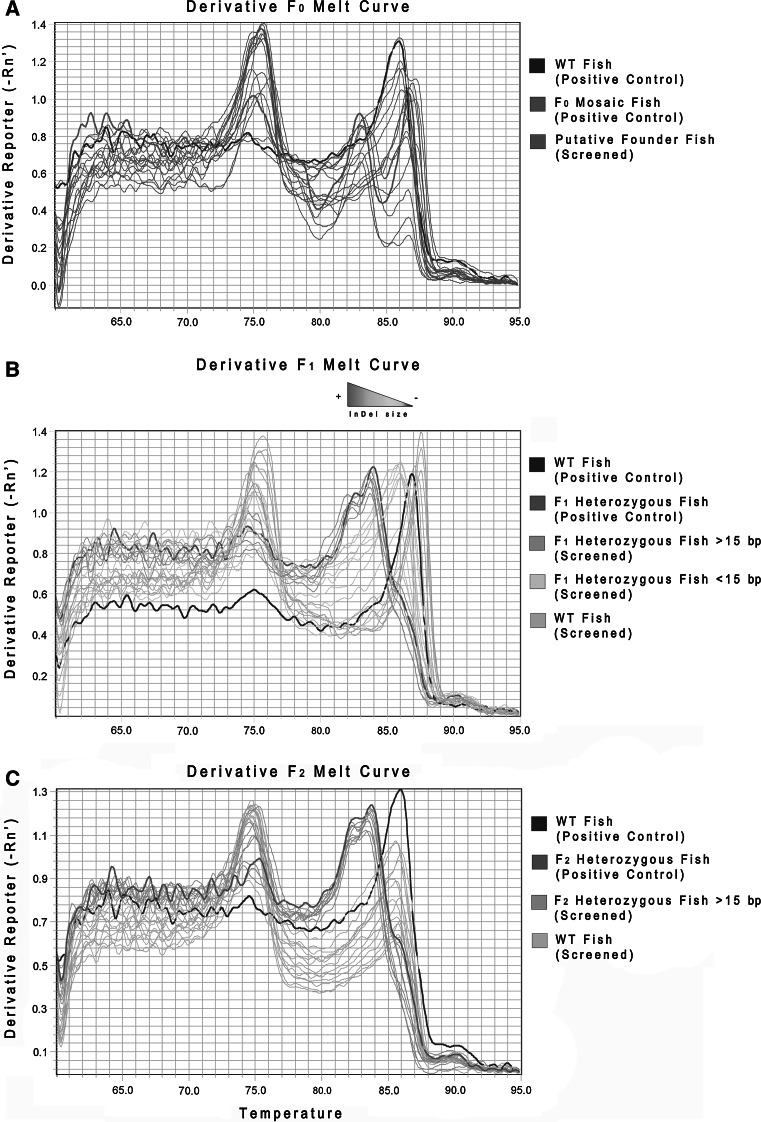
Derivative melting curve profiles. **a** F0 melting curve profiles: the *blue curve* corresponds to a known wt genotype, while the *red* indicates a mosaic fish known from sequencing to be mutated in somatic and germ lines. The *grey* profiles represent the screened microinjected fishes (putative founders) that show heterogeneous curves. **b** F1 melting curve profiles: the *blue curve* corresponds to a known wt genotype used as reference, while the *red* indicates a F_1_ heterozygous fish known from sequencing to be mutated (mutant F_1_ reference). *Dark orange* and *light orange* represent the F_1_ heterozygous fishes showing mutations more (>) or less (<) than 15 bp long, respectively. *Light blue* indicates the wt genotype obtained from the F_1_ screening analysis. **c** F2 melting curve profiles of an outcrossed F_1_ heterozygous fish carrying a >15 bp mutation. The *blue curve* corresponds to a known wt genotype, while the *red* indicates a F_2_ heterozygous fish known from sequencing to be mutated (mutant F_2_ reference). *Dark orange* represents the selected F_2_ heterozygous mutant showing >15 bp mutation. *Light blue* indicates the wt genotype resulted from the F_2_ screening process. The blank control is shown in Additional material Fig. 1 (Color figure online)

In order to identify the founder fish, we first screened the microinjected fishes (F0). As showed in Fig. 1a, two sequenced control fishes were employed as reference: 1) a wt fish showing a single peak in the derivative melting profile (blue curve), and a mosaic fish (F0) that presents a complex melting profile (red curve) with multiple peaks with respect to the wt. Grey curves represent the screened putative founders (mosaic) which show multiple peaks, with a degree of chimerism that may depends on when the genomic mutation occurred during early zygotic cell divisions. To confirm the result of our F0 melting curve analysis, we sequenced the genomic target region from 15 fishes, which showed mutant or wt melting curves. Sequence data confirmed that those fishes with a melting curve similar to the mosaic reference fish (red curve, Fig. 1a) were indeed mutated, while the fishes with a melting curve similar to wt (blue curve, Fig. 1a) were mostly not mutated, except in few cases where we found deletions smaller than 15 bp (Additional material Table 1).

Afterwards, to validate the efficiency of our method, we screened the offspring (F1) of previously identified F0 mosaic fishes crossed with wt (Fig. 1b). Also in this case, we used two sequenced control fishes as reference: a wt (blue curve) and a F1 heterozygous fish (red curve), showing different melting curve profiles (Fig. 1b). The orange curves represent the F1 heterozygous fishes screened, grouped in two different categories based on the InDel size obtained: less than 15 bp (light orange) and more than 15 bp (dark orange). Interestingly, we noticed that the broader the InDels size is, the more the curve is shifted towards lower melting temperature, as shown by the triangle of gradients on the top of Fig. 1b. Those fishes that resulted not mutated from the screening analysis are shown in light blue. The result of the F1 melting analysis was confirmed by sequencing as previously described (Additional material Table 1).

Our method does not show the maximum efficiency in the initial identification of the best mutation to carry on, because the derivative melting curve of mutants with InDels less than 15 bp is not always clearly distinguishable from the wt one. Nevertheless the presented methodology can be used as a preliminary one-step approach for massive screening, in order to restrict the number of embryos to grow up and to focus only on those for the next steps.

However, once the desirable mutation fixed in the carrier fish (F1) has been identified, our methodology is very effective for the F2 screening (Fig. 1c). Since this progeny derives from an outcross of a selected F_1_ heterozygous fish carrying a mutation with more than 15 bp, there is only one possible type of mutant curve with a trend similar to the heterozygous control fish (red curve, Fig. 1c), clearly distinguishable from the wt one used as reference (blue curve). This is due to the fact that the F2 generation is made of 50 % wt (light blue) and 50 % heterozygous fishes carrying the same InDel mutation (orange curves). Also in this case, we confirmed the results by sequencing (Additional material Table 1).

To obtain the stable *knock*-*out* fish line the F3 progeny need to be generated, and we expect that in this case the resulting melting curve would be almost identical to the F2 generation melting profile (red curve in Fig. 1c), given that the InDel mutation is well fixed in the genome at this stage. An alternative scenario could be that the homozygous mutant curve is shifted towards lower temperature values than the heterozygous one. This tendency was already observed for carriers (F_1_) in comparison to wild types. Further experiments are necessary to clarify which hypothesis is correct.

In conclusion, this approach provides a simple, rapid and low-cost protocol for InDels detection, accessible to any research laboratory. This method can be applied to conventional ViiA™7 Real-Time PCR System for qPCR, bypassing in this way the necessity of expensive laboratory equipment. Compared with other screening approaches the presented methodology shows a better advantage versus disadvantage ratio (highlighted in red in Table 1) and can be profitably used in a routinely screening procedure. Moreover, this approach has the potential to be applied for the high-throughput screening in zebrafish as well as in every animal model suitable for genome editing.

**Table 1:** Screening techniques: costs and benefits

Approach	Cost	Equipment	Time	Accuracy	Throughput
Locus DNA sequencing^a^	High	High	Average	High	Average
Fluorescent PCR^b^	High	High	Average	High	High
PAGE-based methods^b^	Low	Low	High	Average	Low
T7 endonuclease I assay^b^	Average	Low	Average	Average	Average
HRM analysis^b^	Low	High	Low	Average	High
Melting Curve analysis^b^	Low	Low	Low	Average	High

It allows the identification of the genomic locus sequence^a^ or the isolation of the carrier fish^b^

## Electronic supplementary material

Supplementary Fig. 1 Blank control. The profile show a predominant peak at 76 °C and two small additional peaks corresponding to higher temperatures. Primer self-annealing probably generates this profile that is clearly different from the one obtained by mutant or wt fishes. Supplementary material 1 (JPEG 1248 kb)

Supplementary Fig. 2 Triplicate derivative melt curves to ensure the reproducibility of the method. Three fishes with different genotypes are shown: **a** wild type; **b** >15 bp heterozygous; **c** <15 bp heterozygous. Supplementary material 2 (JPEG 4918 kb)

Supplementary Table 1 The target regions of 52 zebrafish from three different generations (F_0_, F_1_ and F_2_) were sequenced in order to validate the melting curves analysis results. The contemporary presence of wt and mutated sequences in the same animal is due to the fact that the screened fishes are mosaic (F_0_) or heterozygous (F_1_ and F_2_). On the other hand, the sequencing of fishes with a wt derivative melting curve, in some cases, detected the presence of short InDels (<15 bp) that were not discriminated by our method. Supplementary material 3 (DOC 111 kb)
