# Supplementary figures and images for: A Rapid and Cheap Methodology for CRISPR/Cas9 Zebrafish Mutant Screening
Source: Mol Biotechnol. 2015 Dec 16;58:73–8. doi: 10.1007/s12033-015-9905-y (PMC4709366; doi:10.1007/s12033-015-9905-y)

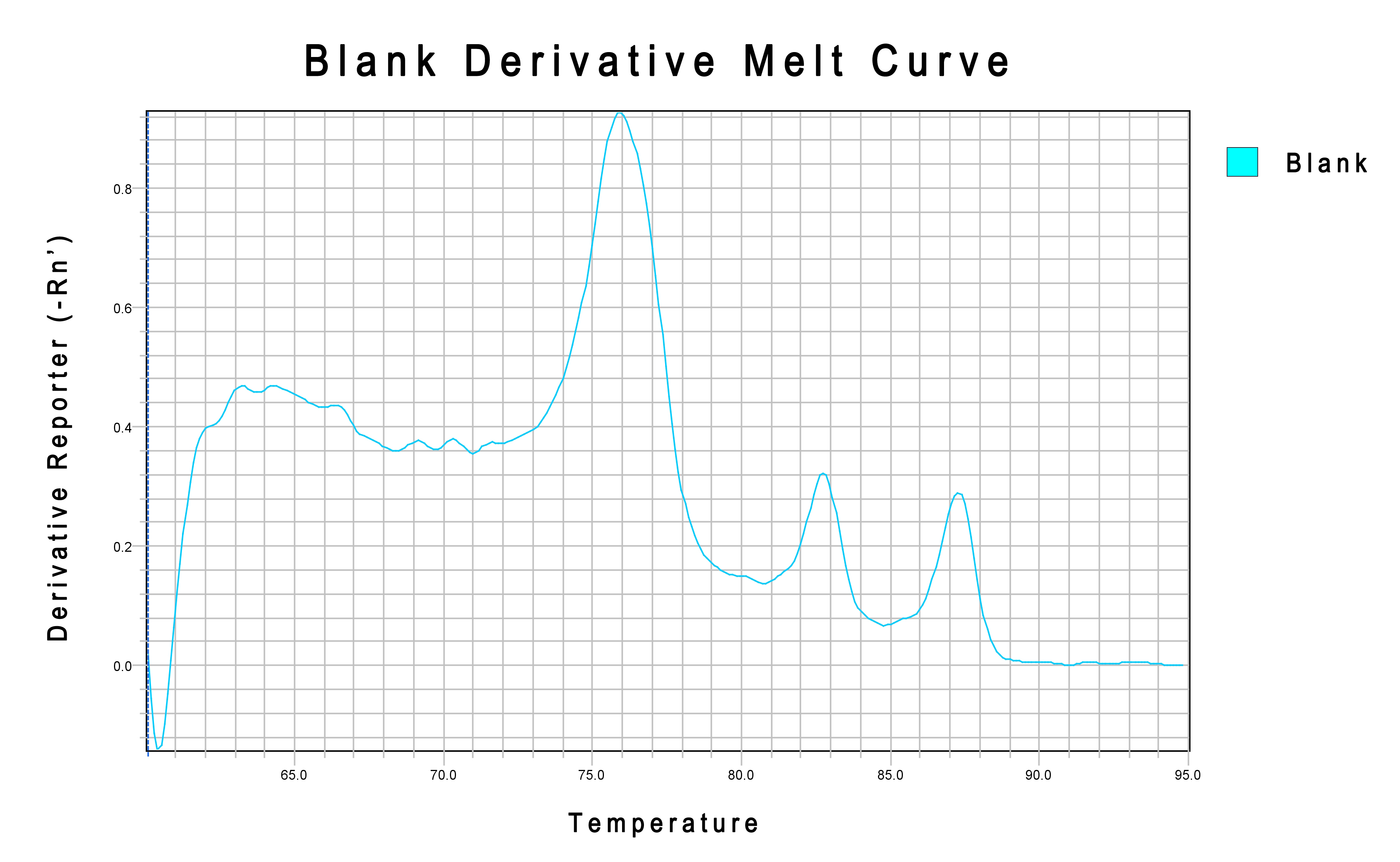

Supplement: Supplementary file 1 — Blank control. The profile show a predominant peak at 76 °C and two small additional peaks corresponding to higher temperatures. Primer self-annealing probably generates this profile that is clearly different from the one obtained by mutant or wt fishes. Supplementary material 1 (JPEG 1248 kb) [file 12033_2015_9905_MOESM1_ESM.jpg]

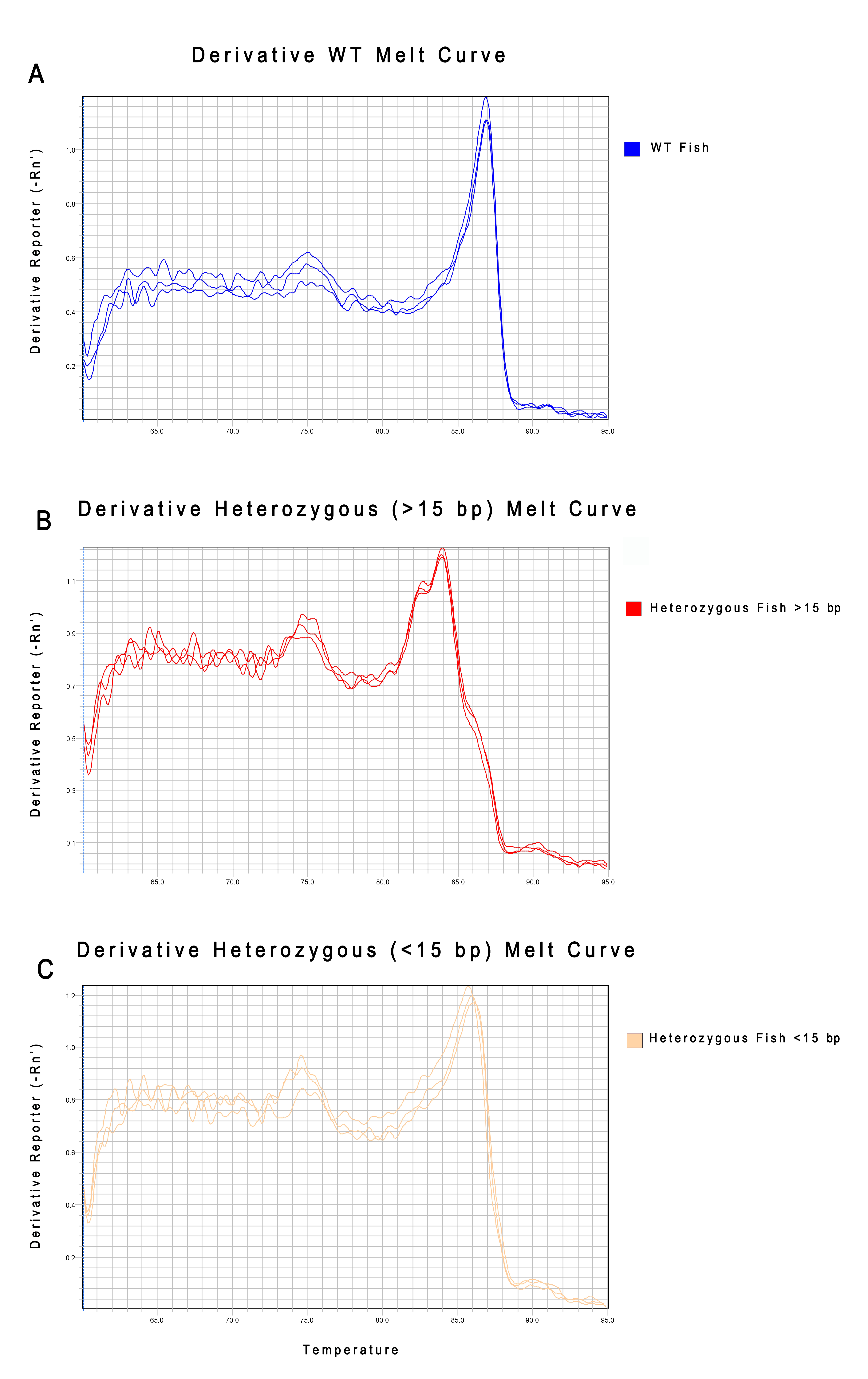

Supplement: Supplementary file 2 — Triplicate derivative melt curves to ensure the reproducibility of the method. Three fishes with different genotypes are shown: a wild type; b >15 bp heterozygous; c <15 bp heterozygous. Supplementary material 2 (JPEG 4918 kb) [file 12033_2015_9905_MOESM2_ESM.jpg]
