# Supplementary material for: A Rapid and Cheap Methodology for CRISPR/Cas9 Zebrafish Mutant Screening
Source: Mol Biotechnol. 2015 Dec 16;58:73–8. doi: 10.1007/s12033-015-9905-y (PMC4709366; doi:10.1007/s12033-015-9905-y)
Supplement: Supplementary file 3 — The target regions of 52 zebrafish from three different generations (F0, F1 and F2) were sequenced in order to validate the melting curves analysis results. The contemporary presence of wt and mutated sequences in the same animal is due to the fact that the screened fishes are mosaic (F0) or heterozygous (F1 and F2). On the other hand, the sequencing of fishes with a wt derivative melting curve, in some cases, detected the presence of short InDels (<15 bp) that were not discriminated by our method. Supplementary material 3 (DOC 111 kb) [file 12033_2015_9905_MOESM3_ESM.doc]

**Additional Table 1: Sequencing results**

| **Screened fishes** | **Mutant from**  **qRT-PCR** | **N° of analysed sequences** | **Resulted wt** | **Resulted mutated** | **Observed mutation.**  **Deletion length (bp)** |
| --- | --- | --- | --- | --- | --- |
| Fish n° 1 (F0) | no | 7 | 5 | 2 | 14  2 |
| Fish n° 2 (F0) | no | 7 | 7 | 0 | -- |
| Fish n° 3 (F0) | yes | 14 | 3 | 11 | 10  27  36  307 |
| Fish n° 4 (F0) | yes | 14 | 4 | 10 | 265  22 |
| Fish n° 5 (F0) | no | 5 | 3 | 2 | 14  3 |
| Fish n° 6 (F0) | yes | 14 | 2 | 12 | 202  14 |
| Fish n° 7 (F0) | yes | 6 | 1 | 5 | 15  49  165 |
| Fish n° 8 (F0) | no | 7 | 5 | 2 | 9 |
| Fish n° 9 (F0) | yes | 6 | 1 | 5 | 36  12  125 |
| Fish n° 10 (F0) | no | 7 | 7 | 0 | -- |
| Fish n° 11 (F0) | yes | 6 | 3 | 3 | 43  414  2 |
| Fish n° 12 (F0) | yes | 6 | 2 | 4 | 16  42  45 |
| Fish n° 13 (F0) | yes | 7 | 2 | 5 | 3  315  42 |
| Fish n° 14 (F0) | yes | 6 | 1 | 5 | 36  14  127 |
| Fish n° 15 (F0) | no | 7 | 7 | 0 | -- |
| Fish n° 16 (F1) | yes | 5 | 1 | 4 | 336 |
| Fish n° 17 (F1) | no | 5 | 4 | 1 | 14 |
| Fish n° 18 (F1) | no | 4 | 4 | 0 | -- |
| Fish n° 19 (F1) | yes | 4 | 1 | 3 | 16 |
| Fish n° 20 (F1) | yes | 3 | 0 | 3 | 414 |
| Fish n° 21 (F1) | no | 5 | 3 | 2 | 2 |
| Fish n° 22 (F1) | yes | 3 | 1 | 2 | 15 |
| Fish n° 23 (F1) | no | 5 | 5 | 0 | -- |
| Fish n° 24 (F1) | no | 5 | 3 | 2 | 15 |
| Fish n° 25 (F1) | yes | 5 | 1 | 4 | 43 |
| Fish n° 26 (F1) | no | 5 | 5 | 0 | -- |
| Fish n° 27 (F1) | yes | 4 | 1 | 3 | 52 |
| Fish n° 28 (F1) | no | 5 | 5 | 0 | -- |
| Fish n° 29 (F1) | yes | 4 | 1 | 3 | 46 |
| Fish n° 30 (F1) | no | 5 | 3 | 2 | 14 |
| Fish n° 31 (F1) | yes | 5 | 1 | 4 | 36 |
| Fish n° 32 (F1) | no | 5 | 4 | 1 | 15 |
| Fish n° 33 (F2) | yes | 5 | 1 | 4 | 43 |
| Fish n° 34 (F2) | yes | 5 | 3 | 2 | 43 |
| Fish n° 34 (F2) | yes | 5 | 2 | 3 | 43 |
| Fish n° 36 (F2) | no | 5 | 5 | 0 | -- |
| Fish n° 37 (F2) | no | 5 | 5 | 0 | -- |
| Fish n° 38 (F2) | yes | 5 | 2 | 3 | 43 |
| Fish n° 39 (F2) | yes | 5 | 0 | 5 | 43 |
| Fish n° 40 (F2) | yes | 5 | 2 | 3 | 43 |
| Fish n° 41 (F2) | yes | 5 | 2 | 3 | 43 |
| Fish n° 42 (F2) | no | 5 | 5 | 0 | -- |
| Fish n° 43 (F2) | no | 5 | 5 | 0 | -- |
| Fish n° 44 (F2) | no | 5 | 5 | 0 | -- |
| Fish n° 45 (F2) | yes | 5 | 2 | 3 | 43 |
| Fish n° 46 (F2) | no | 5 | 5 | 0 | -- |
| Fish n° 47 (F2) | no | 5 | 5 | 0 | -- |
| Fish n° 48 (F2) | yes | 5 | 0 | 5 | 43 |
| Fish n° 49 (F2) | no | 5 | 5 | 0 | -- |
| Fish n° 50 (F2) | yes | 5 | 1 | 4 | 43 |
| Fish n° 51 (F2) | yes | 5 | 2 | 3 | 43 |
| Fish n° 52 (F2) | no | 5 | 5 | 0 | -- |
